# The influence of insulin-related genetic variants on fetal growth, fetal blood flow, and placental weight in a prospective pregnancy cohort

**DOI:** 10.1038/s41598-023-46910-6

**Published:** 2023-11-10

**Authors:** Pauline K. Reim, Line Engelbrechtsen, Dorte Gybel-Brask, Theresia M. Schnurr, Louise Kelstrup, Estrid V. Høgdall, Torben Hansen

**Affiliations:** 1 Faculty of Health Sciences, Novo Nordisk Foundation Center for Basic Metabolic Research, University of Copenhagen, Maersk Tower, Blegdamsvej 3B, 8th Floor, 2200 Copenhagen, Denmark; 2 Department of Gynaecology and Obstetrics, Herlev Hospital, University of Copenhagen, Herlev, Denmark; 3 Psycotherapeutic Outpatient Clinic, Department of Psychiatry, Ballerup Hospital, Ballerup, Denmark; 4 Molecular Unit, Department of Pathology, Herlev Hospital, University of Copenhagen, Herlev, Denmark

**Keywords:** Genetics, Endocrinology, Metabolic disorders, Intrauterine growth

## Abstract

The fetal insulin hypothesis proposes that low birthweight and type 2 diabetes (T2D) in adulthood may be two phenotypes of the same genotype. In this study we aimed to explore this theory further by testing the effects of GWAS-identified genetic variants related to insulin release and sensitivity on fetal growth and blood flow from week 20 of gestation to birth and on placental weight at birth. We calculated genetic risk scores (GRS) of first phase insulin release (FPIR), fasting insulin (FI), combined insulin resistance and dyslipidaemia (IR + DLD) and insulin sensitivity (IS) in a study population of 665 genotyped newborns. Two-dimensional ultrasound measurements with estimation of fetal weight and blood flow were carried out at week 20, 25, and 32 of gestation in all 665 pregnancies. Birthweight and placental weight were registered at birth. Associations between the GRSs and fetal growth, blood flow and placental weight were investigated using linear mixed models. The FPIR GRS was directly associated with fetal growth from week 20 to birth, and both the FI GRS, IR + DLD GRS, and IS GRS were associated with placental weight at birth. Our findings indicate that insulin-related genetic variants might primarily affect fetal growth via the placenta.

## Introduction

It is well established that early life exposures may have lifelong impact on human health, and that impaired fetal growth affects the risk of developing cardiometabolic diseases in adulthood^1–5^. Previous research has demonstrated a U-shaped relation between birthweight and later risk of type 2 diabetes (T2D), indicating that adverse fetal growth can influence the lifelong risk of T2D^6^. The early life programming effects are influenced by both periconceptional, fetal and postnatal factors^7,8^.The role of genetic susceptibility to T2D during the life course is complex and may have several, time-specific effects on human physiology^9^.

Traditionally, maternal glucose levels was considered a key determinant in the variance of birthweight; high birthweight was primarily considered to be a result of maternal hyperglycaemia which caused fetal hyperinsulinemia and low birthweight considered to be a result of maternal malnutrition^10^. However, this theory did not fully explain the increased risk of T2D in low birthweight individuals^11^, and later discoveries revealed that the impact of maternal glucose levels on birthweight is estimated to explain only 3–13% of the variance in birthweight^12,13^**.** A recent study confirmed that fetal insulin is a potent growth factor in utero^14^. As maternal insulin does not cross the placenta, insulin levels in utero are entirely determined by the ability of the fetus to produce and secrete fetal insulin. Thus, the fetal response to intrauterine glucose levels is highly dependent on fetal beta cell function^14,15^.

Fetal growth is affected by both the maternal and the fetal genes^16–18^. The maternal genes affect fetal growth indirectly through effects on the intrauterine environment^16^ and consequently on glucose levels in the fetal circulation. The fetal beta cell function, and hence the fetal response to glucose levels, is affected by variation in the fetal genes^4,14^.

The fetal insulin hypothesis, established in 1998, proposes that low birthweight and T2D are two phenotypes of the same genotype^6,7,11,19^, indicating that genetic variants of insulin-related traits can restrict growth in fetal life and increase the risk of T2D in adulthood by affecting insulin release and sensitivity in utero as well as later in life. Genetic studies have tried to disentangle the causal mechanisms of the association between birthweight and subsequent risk of T2D. The first approaches to understand causal mechanisms originated from studies of monogenic diabetes, in which mutations in the fetal glucokinase gene (GCK) were shown to cause a reduction in both insulin-mediated fetal growth in utero and hyperglycaemia after birth^20^. In addition, studies have shown that monogenic disorders, which impair glucose-sensing, lower insulin secretion, or increase insulin resistance, are associated with impaired fetal growth^11, 14, 21^.

Studies to support a role for polygenic effects came from epidemiological research showing that maternal diabetes were associated with higher birthweight^22^, while paternal diabetes or prediabetic conditions (e.g., preclinical insulin resistance) was associated with lower birthweight^22^. These studies not only suggested that the genetic contribution to birthweight was influenced by the parental genes, but also indicated that genetics may play a crucial role in understanding the link between birthweight and later T2D^23,24^.

A study using data from the UK Biobank investigated the effect of paternal diabetes on offspring birthweight and found that birthweight is one of the mediating factors in the linkage between paternal diabetes and increased risk of T2D in the offspring^22^. A plausible explanation of this could be transmission of genetic variants from father to fetus related to both lower birthweight and increased risk of T2D. In 2018 Udler et al. investigated the effect of T2D risk alleles on birthweight and found significant associations with variants related to both beta cell function, proinsulin secretion and insulin resistance^25^.

Investigating the effects of genetic determinants of insulin release and sensitivity on fetal growth throughout pregnancy may provide unique insights into the role of fetal insulin and might clarify the connection between two epidemiological and genetically related phenotypes; low birthweight and T2D in adulthood.

The overall aim of this study was to investigate the underlying genetic mechanisms linking fetal growth and T2D and expand the basis of the fetal insulin hypothesis. We aimed to test the associations of fetal genetic risk scores (GRS) constructed by genetic variants related to insulin release and insulin sensitivity with fetal weight estimates and fetal blood flow measurements at several timepoints during pregnancy as well as weight measured at birth. Furthermore, we aimed to test the associations between the genetic variants and placental weight.

## Results

### Demographic characteristics

Prenatal data from 665 newborns were included in the analyses. The mothers had a mean ± SD age of 30.2 ± 4.7 years and a pre-pregnancy BMI of 24.8 ± 4.8 kg/m^2^. The newborns had a mean birthweight of 3594.0 ± 464.7 g and were born at a mean gestational age of 279.6 ± 9.1 days^26^. The mean placental weight measured at birth was 648.0 ± 138.7 g (Table 1).

**Table 1:** Demographic characteristics of pregnancies (n = 665).

Variable	Mean (SD)
Maternal age (years)	30.2 (4.7)
Maternal pre-pregnancy BMI (kg/m^2^)	24.8 (4.8)
Birth weight (g)	3594 (464.7)
Gestational age at birth (days)	279.6 (9.1)
Placental weight at birth (g)	648 (138.7)

Information on maternal age and pre-pregnancy BMI is from inclusion in the Roskilde cohort. All numbers are given in mean and standard deviations (SD).

### Associations between genetic risk scores for insulin-related traits and fetal growth

Intrauterine growth was modelled from 20 weeks of gestation to birth and displayed a curvilinear trajectory.

The GRS for first phase insulin release (FPIR) was associated with fetal growth from week 20 to 32 with a proportional weight gain of 5.84e−05 (95%CI: (− 1.47e−05; 1.32e−04) percentage points/allele/day (*p* = 0.043, Table 2). When including weight measured at birth in the linear mixed model the association was accentuated, showing a per allele effect of 8.75e−05 (95% CI: − 2.60e–05; 2.01e−04) percentage points per day (*p* = 0.030, Table 2).

**Table 2:** Associations between unweighted genetic risk scores (GRS) for insulin secretion and sensitivity-related traits and fetal growth.

Trait	Fasting insulin GRS (FI)		First phase insulin release GRS (FPIR)		Insulin resistance and dyslipidaemia GRS (IR + DLD)		Insulin sensitivity GRS (IS)	
	β (95% CI)	P	β (95% CI)	*P*	β (95% CI)	P	β (95% CI)	*P*
Fetal weight (g) (incl. birth weight)	4.63e−05 (− 4.27e−05; 1.35e−04)	0.518	8.75e−05 (− 2.60e−05; 2.01e−04)	**0.030**	7.58e−06 (− 6.28e−05;7.80e−05)	0.617	9.48e−05 (− 8.71e−05; 2.77e−04)	0.293
Fetal weight (g) (excl. birth weight)	3.52e−05 (− 2.20e−05; 9.23e−05)	0.147	5.84e−05 (− 1.47e−05; 1.32e−04)	**0.043**	3.25e−06 (− 4.20e−05; 4.85e−05)	0.316	3.77e−05 (− 7.93e−05; 1.55e−04)	0.300
Occipitofrontal diameter (mm)	4.87e−07 (− 1.52e−06; 2.49e−06)	0.443	2.05e−07 (− 2.36e−06; 2.76e−06)	0.840	1.17e−06 (− 4.12e−07; 2.75e−06)	0.086	2.37e−06 (− 1.72e−06; 6.46e−06)	0.281
Biparietal diameter (mm)	1.71e−06 (− 3.99e−08; 3.47e−06)	**0.035**	8.91e−07 (− 1.35e−06; 3.14e−06)	0.641	4.26e−07 (− 9.61e−07; 1.81e−06)	0.653	1.28e−06 (− 2.32e−06; 4.88e−06)	0.544
Abdominal circumference (mm)	3.45e−06 (− 2.60e−06; 9.49e−06)	0.272	6.55e−06 (− 1.16e−06; 1.43e−05)	0.094	6.61e−07 (− 4.12e−06; 5.44e−06)	0.506	4.40e−06 (− 7.96e−06; 1.68e−05)	0.296
Femur length (mm)	4.09e−07 (− 6.83e−07; 1.50e−06)	0.377	1.57e−06 (1.87e−07; 2.97e−06)	**0.026**	2.01e−07 (− 6.62e−07; 1.06–06)	0.648	4.06e−07 (− 1.83e−06; 2.64e−06)	0.489

Fetal growth was modelled using linear mixed regression. Data is presented as raw parameter estimates (β) and estimates are transformed to the original scale with respective 95% confidence intervals. Beta values represent the additive per allele effect of the GRS on change in daily proportional weight gain. All traits are measured by ultrasound during pregnancy at 20, 25 and 32 weeks. Birthweight was measured at birth.

Significant values are in bold.

The GRS for fasting insulin (FI) was associated with biparietal diameter, but only the GRS for FPIR associated significantly with overall fetal growth (Table 2).

### Associations between genetic risk scores for insulin traits and placental weight

The GRSs for fasting insulin (FI) and combined insulin resistance and dyslipidaemia (IR + DLD) were both negatively associated with placental weight at birth (*p* = 0.048 and *p* = 0.024, respectively), whereas the GRS for insulin sensitivity (IS) was positively associated with placental weight (*p* = 0.0014, Table 3).

**Table 3:** Associations between unweighted genetic risk scores (GRS) for insulin secretion and sensitivity-related traits with placental weight at birth and fetal blood flow measures.

Trait	Fasting insulin GRS		First phase insulin release GRS		Insulin resistance GRS		Insulin sensitivity GRS	
	β (95% CI)	*P*	β (SD)	*P*	β (SD)	*P*	β (SD)	*P*
Placental weight at birth (g)	− 2.04 (− 4.31; 0.22)	**0.048**	0.62 (− 2.30; 3.54)	0.349	− 1.82 (− 3.61; − 0.04)	**0.024**	7.44 (2.88; 12.0)	**0.0014**
Placental ratio (placental weight/ birth weight)	− 0.0008 (− 0.0017; 0.0002)	0.117	− 0.0007 (− 0.002; 0.0005)	0.272	− 0.0002 (− 0.0009; 0.0006)	0.675	0.0009 (− 0.001; 0.003)	0.338
Birth weight (g)	1.185 (− 11.4716; 13.8410)	0.854	15.8209 (− 0.2251; 31.8670)	0.051	− 5.4828 (− 15.4618; 4.4962)	0.236	13.5481 (− 12.2277; 39.3241)	0.348
Small for gestational age at birth (SGA)	− 0.0062 (− 0.1110; 0.0985)	0.907	0.0148 (− 0.1194; 0.1491)	0.829	0.0408 (− 0.0429; 0.1246)	0.339	0.0254 (− 0.1866; 0.2375)	0.814
Large for gestational age at birth (LGA)	− 0.0521 (− 0.2143; 0.1100)	0.528	− 0.1267 (− 0.3260; 0.0726)	0.213	− 0.0936 (− 0.2137; 0.0266)	0.127	0.0703 (− 0.2593; 0.3999)	0.676
Umbilical flow (PI)	0.002 (0.001; 0.005)	0.209	0.001 (− 0.003; 0.005)	0.615	0.0007 (− 0.002; 0.003)	0.591	0.0004 (− 0.006; 0.007)	0.957
Middle cerebral artery flow (PI)	0.0052 (− 0.024; 0.035)	0.797	− 0.0179 (− 0.056; 0.020)	0.345	− 0.0267 (− 0.051; − 0.002)	**0.047**	0.042 (− 0.018; 0.103)	0.191

Placental weight, placental ratio (placental weight/birthweight), and birthweight were modelled using linear regression. SGA and LGA were modelled using logistic regression. Flow parameters were modelled using generalized linear regression. Data are presented as raw parameter estimates (β) and estimates are transformed to the original scale with respective 95% confidence intervals. SGA indicates a birthweight less than 10th percentile for the gestational age. LGA indicates a birthweight above the 90th percentile for the gestational age.

Significant values are in bold.

**Figure 1:**
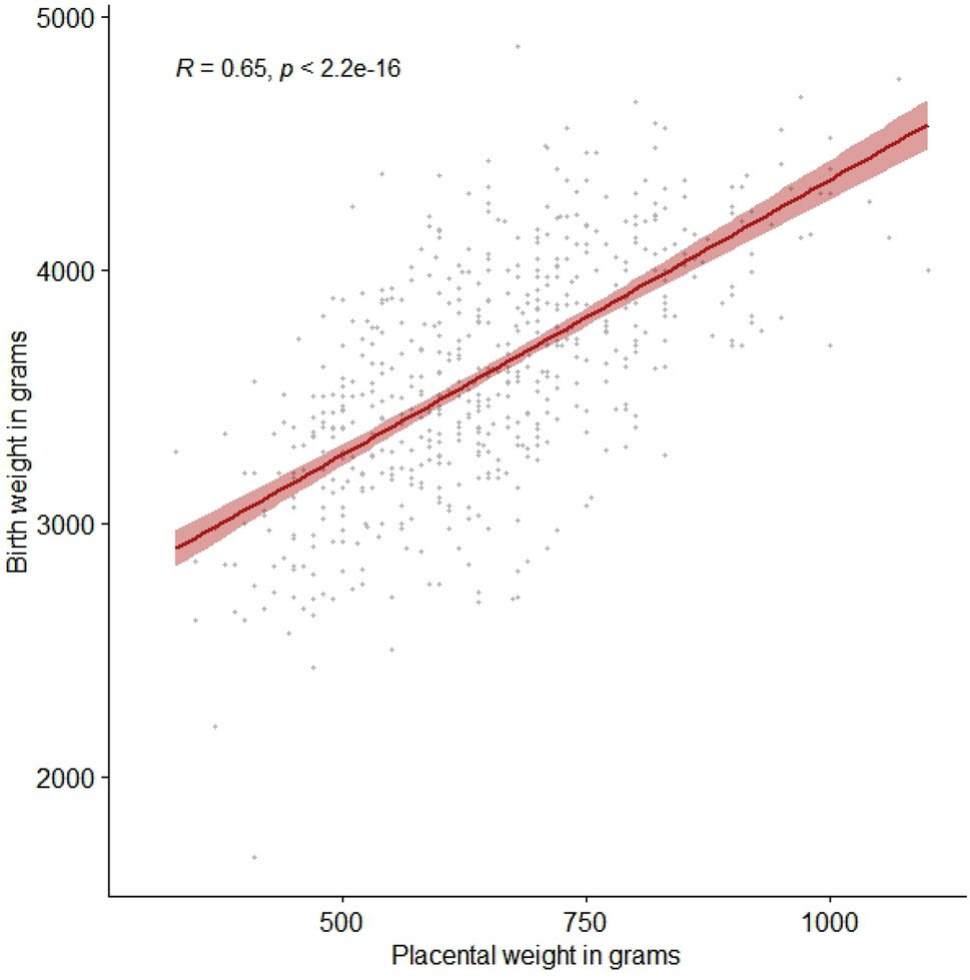
Pearson correlation test between birthweight and placental weight. R represents the Pearson correlation coefficient.

Furthermore, there was a strong correlation between placental weight and weight at birth with a Pearson correlation coefficient of 0.65 (95% CI 0.60–0.69, *p* = 2.2 × 10e−16), illustrated in Fig. 1.

### Genetic risk scores for insulin traits and association with fetal flow parameters

A borderline significant negative association was found between GRS for IR + DLD and the fetal middle cerebral artery (MCA) flow (*p* = 0.047, Table 3).

No other significant associations between GRSs for the insulin traits and fetal flow parameters in pregnancy were found (Table 3).

**Figure 2:**
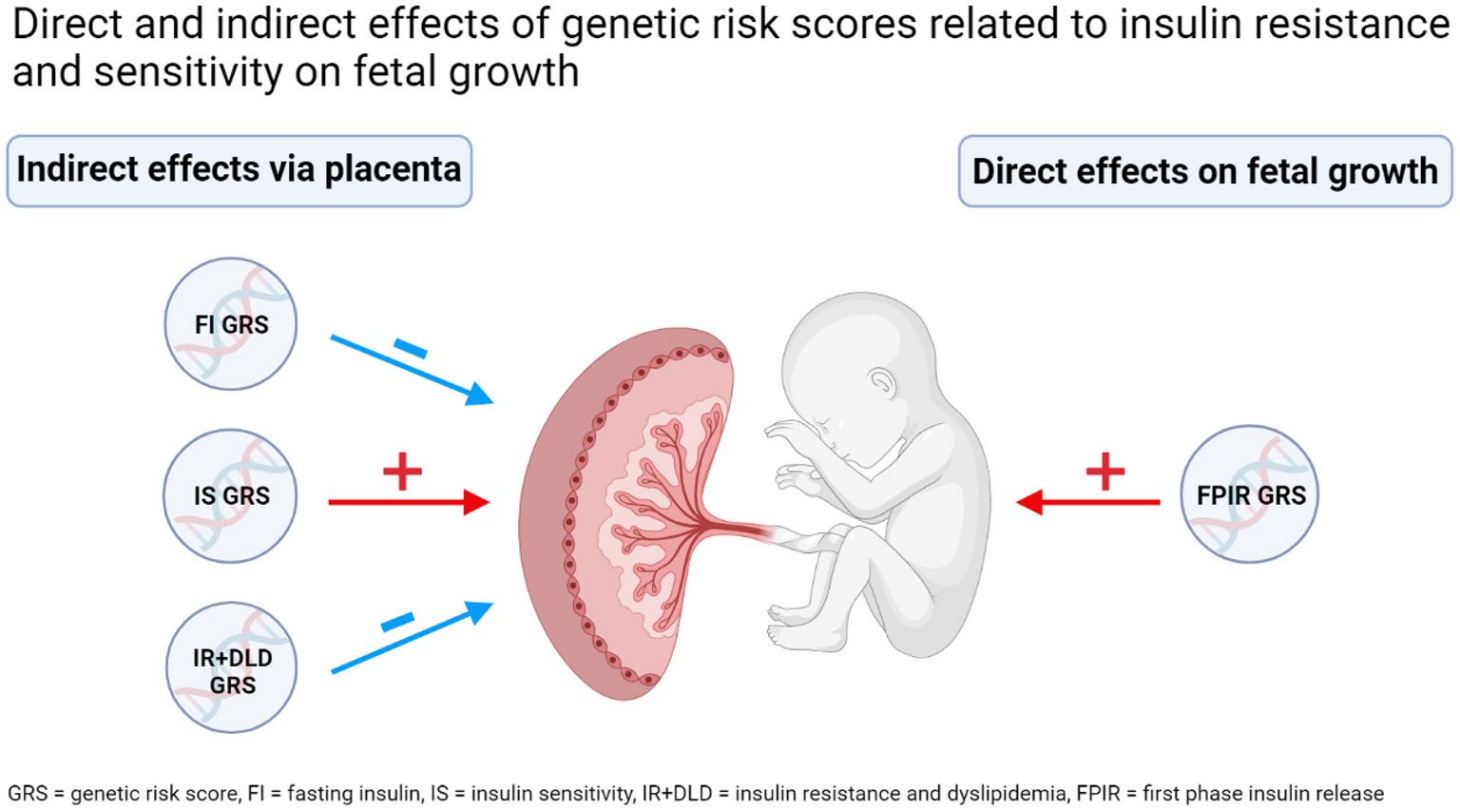
Graphical summary of findings. Created by the first author (PR) with BioRender.com.

A graphical summary of the findings is presented in Fig. 2.

Association analyses with weighted GRSs are available in Supplementary (Table S1 and S2).

## Discussion

This study has addressed the fetal insulin hypothesis^11^ in a study population of 665 genotyped newborns by investigating the effect of genetic variants for insulin-related traits defined by previous GWASs^27–31^ on fetal growth and fetal blood flow as well as birthweight and placental weight.

We demonstrate that genetic variants associated with FPIR are associated with increased fetal growth from week 20 to birth. In addition, we demonstrate that genetic variants associated with FI, IR + DLD, and IS have variable effects on placental weight indicating that the underlying genetic determinants of insulin release and sensitivity may have a substantial impact on placental physiology, that could mediate effects on fetal growth.

Previous studies have mainly used birthweight as a surrogate measure of fetal growth during pregnancy^7,32–34^. Weight at birth is determined by fetal growth throughout pregnancy which is affected by multiple factors related to the intrauterine environment, physiology, and genetics in a complex interplay between maternal and fetal factors^35^. These factors might act in specific timeframes as opposed to through the entire pregnancy^26,35^. In this study, we evaluated effects of genetic variants on overall fetal growth from 20 weeks of gestation to birth as opposed to weight at birth exclusively, enabling a unique insight into fetal growth trajectories during pregnancy, which can potentially provide better knowledge on the influence of fetal genetic variants for insulin-related traits.

Several genetic variants affecting birthweight have been shown to also strongly associate with T2D risk^25,33,36^ providing evidence that the association between birthweight and T2D risk has a distinct genetic component. A more recent study found that several genetic variants identified to regulate fetal growth throughout pregnancy were associated with T2D^26^. Today, at least 11 variants known to be associated with T2D are also affecting birthweight, 10 of which decrease birthweight^4^. The results of this study expand the basis of the fetal insulin hypothesis by linking genetic variants involved in insulin release and sensitivity to fetal growth throughout pregnancy and placental weight at birth.

A study by Hughes et al. found that the effect of fetal genetic variants associated with birthweight was independent of maternal fasting glucose levels^37^. Furthermore, they reported that in pregnancies with elevated maternal fasting plasma glucose levels the frequency of LGA (large-for-gestational age) was 31% for offspring with the highest tertile GRS for birthweight compared with a frequency of only 14% for offspring with the lowest tertile GRS for birthweight^37^. This suggests that fetal genetics have a major impact on fetal growth and that fetal genetics might determine to which degree the fetus is affected by the intrauterine environment.

We found that the fetal GRS for FPIR was positively associated with fetal growth from week 20 to 32, supporting the theory that fetal growth is dependent on fetal beta-cell function^15^, which is affected by variation in the fetal genes^4^. The association was stronger when birthweight was added to the model.

Fetal insulin is one of the key determinants of fetal growth and has been shown to account for nearly half of the birthweight at term^14^. Fetal insulin acts mainly in the third trimester—from gestational week 28 to birth^10,11^—and therefore the effects of GRSs of insulin-related traits may be more substantial in the third trimester compared to the first and second trimester. This could explain why the association between the GRS of FPIR and fetal growth is accentuated when birthweight is added to the model. In this study we only included one measurement in the third trimester (week 32). Future studies might clarify the effect of insulin-related variants on fetal growth by including more measurements on fetal growth in the third trimester, although ultrasound measurements closer to term are much less precise than measurements performed in the first and second trimester^38^.

A GWAS on placental weight by Beaumont et al.^39^ support the theory that fetal insulin plays a role in regulating placental weight^39^. Our study revealed a positive association between the GRS for IS and placental weight at birth. In addition, the GRSs for FI and IR + DLD were negatively associated with placental weight at birth. These findings align with the rationale of the fetal insulin hypothesis^4^. A high GRS for IS may enhance the function of fetal insulin and thereby increase the ability of the fetus to metabolize glucose and grow. Similarly, an increased GRS for IR + DLD might impair the fetal glucose metabolism and restrict fetal growth.

We found a good correlation between placental weight and birthweight, which is in concordance with previous studies^40,41^. Physiologically the fetal ability to grow is determined by the supply of nutrients and oxygen for the fetus during pregnancy, which is regulated by the maternal and fetal metabolism as well as the placental and fetal blood flow. The growth potential of the fetus is determined by a wide range of factors including the fetal genes, which affect fetal growth directly and indirectly through effects on the intrauterine environment and placental physiology^32,35^. Hence, the effects of genetic variants encoding insulin traits on fetal growth could be mediated via the placenta.

Restriction of fetal growth can cause impaired fetal blood flow, which can be detected by ultrasound as early as 24 weeks of gestation^42^. Our study showed a borderline association between one of the GRSs (insulin resistance and dyslipidaemia) and a single flow parameter (MCA). No associations between MCA flow and other GRSs were identified. Moreover, we did not find associations between any of the GRSs and flow in the umbilical artery. This might be because the genetic determinants of flow parameters are not captured by genetic variants of insulin-related traits but rather by variants related to other cardiometabolic traits known to be associated with T2D risk.

This study has some limitations. Estimating fetal weight by the Hadlock Formular based on ultrasound examinations is subject to inaccuracies particularly in the third trimester and increasing towards term^38^. We did not account for intra- or interobserver variations in measurements, but used the same few experienced sonographers all through the study period to limit variations.

**Figure 3:**
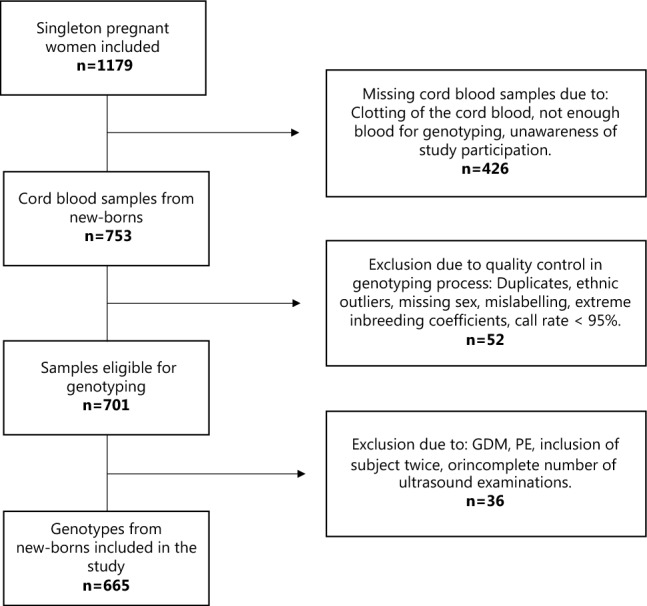
Flowchart of Study Population. GDM = Gestational Diabetes Mellitus, PE = Pre-eclampsia.

Umbilical cord blood samples need to be taken directly after birth and is therefore often missed in a delivery ward with other important and time sensitive tasks. Still, a subset of 753 newborns out of nearly 1200 pregnancies were included in the neonatal study population (Fig. 3). The population used in this study consists of 665 genotyped newborns (Fig. 3), which for genetic studies is a quite small sample size that might not provide sufficient power to detect statistically significant associations. However, we did find significant associations—all of which are in line with previous findings and in agreement with theories of how measures of fetal insulin sensitivity and release might affect fetal growth^4, 14^. We used previously identified genetics variants from GWASs in adults and applied them to calculate fetal GRSs. The effect-size of each variant in fetuses is not known and might differ substantially from the effect sizes in adults and vary throughout pregnancy. Therefore, we chose to calculate unweighted GRSs. Association analyses with weighted GRSs are available in Supplementary material (Table S1) and do not differ considerably from the results presented.

As this study investigated the effects of fetal GRSs constructed by genetic variants from genotyped newborns, we were not able to adjust for maternal genetic effects. Calculating the maternal GRSs of insulin-related traits in this study population and compare their effects on fetal growth, fetal blood flow and placental weight to the effects of the fetal GRSs would contribute to the understanding of the complex direct (fetal) and indirect (maternal) genetic effects.

In conclusion, this study indicates that genetic variants related to insulin release and sensitivity inherited by the fetus may affect fetal growth directly (GRS for FPIR) and indirectly (GRS for FI, IS and IR + DLD) through effects on placental physiology during pregnancy. These findings strengthen the link between low birthweight and the subsequent risk of T2D in the offspring and add to the growing knowledge of genetic determinants to play a substantial role in this association.

Our findings contribute to a future facilitation of early life identification of individuals at increased risk of T2D, to whom preventative strategies to postpone disease development and/or improve disease prognosis could be highly beneficial.

## Methods

### Study population

Pregnant women (> 18 years of age) attending a routine first trimester ultrasound examination at Roskilde hospital in Denmark at gestational age 12–14 weeks were invited to participate in this study. The study population has previously been described in detail^26,43,44^. A total of 1179 singleton pregnant women fulfilling inclusion criteria were included in the study (Figure S1), and all gave oral and written informed consents prior to enrolment. Participants were invited for additional ultrasound examinations at gestational age (GA) 20, 25 and 32 to assess fetal malformations (GA 20), fetal growth and fetal blood flow. Birthweight and placental weight were registered at birth by trained hospital personal. Cord blood samples were collected at birth in 753 out of 1179 pregnancies*.* However, after exclusion of genotypes that did not pass genotyping quality control (n = 52) and exclusion of cases with gestational diabetes, pre-eclampsia, incomplete number of ultrasound examinations, inclusion of pregnancy twice, or presence of an a-cardiac twin (n = 36), a total of 665 genotypes from newborns were included in the analyses of this study (Fig. 3).

### Ultrasound scans

Ultrasound scans were performed by trained sonographers or medical doctors at gestational week 12, 20, 25 and 32 on the following ultrasound equipment: Voluson E8, Voluson Expert 730, Voluson e, and Voluson Logic 7 (General Electric Company, Fairfield, CT, USA). Astraia software version 1.12.9 (Astraia software gmbh, München, Germany) was used. Gestational age was based on ultrasound measurements of the fetal crown–rump length (CRL) at the nuchal translucency scan (12 weeks).

### Fetal growth measurements

Fetal weight was assessed based on two-dimensional ultrasound scans which included measurements of abdominal circumference (AC) in milli meters (mm), bi-parietal diameter (BPD) in mm, occipito-frontal diameter (OFD) in mm and length of the femur bone (FL) in mm.

Fetal weight in grams was calculated based on the Hadlock formula^42^:

$$\begin{aligned} {\text{Weight}}({\text{g}}) & = 10^\wedge(1.326 - 0.00326 \times {\text{AC}} \times {\text{FL}}/100) + (0.0107 \times {\text{HC}}/10) \\ & \quad + (0.0438 \times {\text{AC}}/10) + (0.158 \times {\text{FL}}/10)). \\ \end{aligned}$$

Abnormal fetal growth was defined at birth as either small-for-gestational-age (SGA) or large-for-gestational-age (LGA). SGA was defined as a birthweight less than 10th percentile for gestational age. LGA was defined as a birthweight above 90th percentile for gestational age^45,46^.

### Fetal blood flow measurements

Fetal flow in the umbilical artery and middle cerebral artery (MCA) were addressed by pulsatility index (PI) measured in week 20, 25 and 32 of pregnancy for the umbilical artery and week 25 and 32 for the MCA. PI was calculated by subtracting the end-diastolic velocity (EDV) from the peak systolic velocity (PSV) and then dividing by the time-averaged (mean) velocity (TAV) at each scan: PI = (PSV − EDV)/TAV.

All flow measurements were carried out with an insonation angle of < 30 degrees.

### Pregnancy outcomes

Information on type of delivery (vaginal or c-section), gestational age at delivery, birthweight, placental weight, and sex of the newborn as well as medical conditions during pregnancy (e.g., gestational diabetes and pre-eclampsia) were retrieved form maternal medical records.

### Anthropometric measurements of the mother

Demographic data including maternal age and pre-pregnancy BMI were reported at the first visit. Additionally, information on smoking and medical conditions, such as diabetes, was registered at inclusion as well as during pregnancy.

### Biochemical measures

At delivery cord blood was sampled for fetal genotyping. It was not possible to obtain cord blood at all deliveries due to the following reasons: Clotting of the cord blood, not enough blood for sampling, or unawareness of participation in the study from the midwife/doctor attending the delivery. Out of 1179 deliveries a total of 753 cord blood samples were retrieved and kept in a − 80 °C freezer until DNA extraction (Fig. 3).

### Genotyping and QC

DNA extracted from 753 cord blood samples were used for fetal genotyping using the Illumina Infinium HumanCoreExome Beadchip platform (Illumina, San Diego, CA). Genotypes were called using the Genotyping module (version 1.9.4) of GenomeStudio software (version 2011.1, Illumina). Genotype imputations were conducted using the Haplotype Reference Consortium (HRC, release 1.1). We excluded, duplicates, ethnic outliers, and samples with extreme inbreeding coefficients, mislabelled or missing sex, or call rate < 95%, leaving 701 samples which passed all quality control criteria. Cases with maternal medical conditions during pregnancy or incomplete study attendance were excluded, leaving 665 newborns with available genotype data to be included in the final analyses (Fig. 3).

### Genetic risk scores

The applied genetic risk scores (GRSs) were selected based on prior GWAS-identified genetic variants associated with insulin levels, insulin sensitivity or insulin resistance in adult populations. Four GRSs were constructed in the study population of 665 genotyped newborns: (1) fasting insulin (FI) GRS, (2) first phase insulin release (FPIR) GRS, (3) insulin resistance and dyslipidaemia (IR + DLD) GRS, and (4) insulin sensitivity (IS) GRS.

The FI GRS was constructed by 17 loci identified by Scott et al.^27^ and 2 loci identified by Dupuis et al.^28^ of which all 19 loci were shown to be associated with fasting insulin at a genome-wide significant level. The FPIR GRS was constructed by 30 loci identified by Wood et al.^29^ which all reached genome-wide significance in their association with levels of peak insulin during an intravenous glucose tolerance test (IVGTT). The IR + DLD was constructed of 53 loci associated with BMI-adjusted fasting insulin in combination with lower HDL cholesterol and higher LDL levels identified in a GWAS by Lotta et al.^30^ The IS GRS was constructed by 23 loci associated with insulin sensitivity index identified in a GWAS by Walford et al.^31^.

For variants that were not directly genotyped and not imputed, we selected proxies (*r*^2^ ≥ 0.90) for the loci used in the different GRSs. We constructed the GRSs as unweighted GRSs by summing the dosages of alleles associated with the given insulin traits stated above. This approach was considered the most accurate since a weighted GRS would have been based on GWAS-identified effects sizes of variants in adults, which may not be transferable to fetuses.

The GRSs were tested for normal distribution prior to analyses.

### Statistical analysis

After quality control of genotyping results and exclusion of ineligible study subjects a total of 665 newborns with available genotype data were included in the analyses (Fig. 3).

Data analysis was performed in R version 4.1.1 (https://www.r-project.org/). Two-tailed testing was used, and statistical significance was defined as a two-sided p-value below 0.05.

### Fetal growth

To model fetal intrauterine growth we used mixed linear regression models, as reported previously^26^. We used mixed linear regression with an interaction with a quadratic time term adjusted for effects of fetal sex and maternal pre-pregnancy BMI, and interactions between these covariates and the quadratic time term.

$${\text{weight}}\sim {\text{GA }} + {\text{GA}}^{{2}} + {\text{GA}}^{{2}} \times {\text{GRS }} + {\text{GA}}^{{2}} \times {\text{Sex }} + {\text{GA}}^{{2}} \times {\text{BMI}}$$

Fetal weight in grams was log-transformed prior to analysis to address the issue of increasing heteroscedasticity of weight measurements during pregnancy. Models were fitted by restricted maximum likelihood using a Gaussian spatial structure to model the correlation between temporally non-equidistant weight estimates. Model assumptions were assessed visually by inspection of normal probability plots and residual plots.

To assess the impact of the individual genetic variants on overall intrauterine growth, similar conditional growth models with and without adjustment for fetal sex and maternal pre-pregnancy BMI were fitted.

We tested the effect of unweighted GRSs for fasting insulin, first-phase insulin release, combined insulin resistance and dyslipidaemia, and insulin sensitivity on intrauterine growth using mixed linear regression models.

The effects of GRSs for insulin-related traits on fetal growth and birthweight were assessed using linear mixed models. In one model we fitted with the measurements of fetal growth at gestational age 20, 25 and 32 and in the other model with measurements of fetal growth at gestational age 20, 25 and 32 and measurements of birthweight to assess whether birthweight was the sole driver of any identified associations.

Birthweight percentiles were calculated in all newborns. The associations between GRSs for insulin-related traits and abnormal fetal growth—defined as a birthweight < 10th percentile (SGA) or > 90th percentile (LGA)—were assessed using logistic regression models adjusted for maternal pre-pregnancy BMI, maternal age, fetal sex, and gestational age.

### Placental weight, birthweight, and placental ratio

Linear regression models adjusted for fetal sex, maternal pre-pregnancy BMI, maternal age and gestational age at birth were used to address associations between the GRSs and placental weight, birthweight, placental ratio. Placental ratio is the placental weight relative to birthweight (placental weight/birthweight).

We tested for correlation between birthweight and placental weight at birth by use of the Pearson correlation (parametric correlation test).

### Flow parameters

Pulsatility index in fetal vessels (MCA and umbilical artery) were addressed by generalized linear models adjusted for maternal pre-pregnancy BMI, and gestational age.

The collection and analyses of data in this study were approved by the Danish Regional Ethical Committee of Region Zealand (SJ-55, SJ347) and the Capital Region (H-22031782) and by the Danish Data Protection Agency. The study was conducted in accordance with the principles of the Helsinki Declaration and all methods were performed in accordance with relevant guidelines and regulations.

### Supplementary Information

Supplementary Information.

## Data Availability

The authors confirm that, for approved reasons, some access restrictions apply to the data underlying the findings in this study. All data are available from the Novo Nordisk Foundation Center for Basic Metabolic Research, Section of Human Variations and Functional Genomics, University of Copenhagen. Should data be requested, corresponding author, Torben Hansen, may be contacted.

## Abbreviations

T2D: Type 2 diabetes
GDM: Gestational diabetes
IVGTT: Intravenous glucose tolerance test
GWAS: Genome-wide association study
GRS: Genetic risk score
FPIR: First phase insulin release
FI: Fasting insulin
IR + DLD: Insulin resistance and dyslipidaemia
IS: Insulin sensitivity
CRL: Crown-rump-length
BPD: Bi-parietal diameter
OFD: Occipito-frontal diameter
AC: Abdominal circumference
FL: Femur length

## References

[1] Barker D (1991). The intrauterine origins of cardiovascular and obstructive lung disease in adult life. The Marc Daniels Lecture 1990. J. R. Coll. Physicians Lond..

[2] Barker D, Martyn C (1992). The maternal and fetal origins of cardiovascular disease. J. Epidemiol. Community Health.

[3] Smith CJ (2016). The impact of birth weight on cardiovascular disease risk in the Women's Health Initiative. Nutr. Metab. Cardiovasc. Dis..

[4] Hughes AE, Hattersley AT, Flanagan SE, Freathy RM (2021). Two decades since the fetal insulin hypothesis: What have we learned from genetics?. Diabetologia.

[5] Martin-Gronert MS, Ozanne SE (2012). Mechanisms underlying the developmental origins of disease. Rev. Endocr. Metab. Disord..

[6] Harder T, Rodekamp E, Schellong K, Dudenhausen JW, Plagemann A (2007). Birth weight and subsequent risk of type 2 diabetes: A meta-analysis. Am. J. Epidemiol..

[7] Hales C (1991). Fetal and infant growth and impaired glucose tolerance at age 64. BMJ.

[8] Barres R, Zierath JR (2016). The role of diet and exercise in the transgenerational epigenetic landscape of T2DM. Nat. Rev. Endocrinol..

[9] Zanetti D (2018). Birthweight, type 2 diabetes mellitus, and cardiovascular disease: Addressing the barker hypothesis with Mendelian randomization. Circ. Genomic Precis. Med..

[10] Pedersen, J. Diabetes and pregnancy: blood sugar of newborn infants. PhD. thesis. *Copenhagen: Danish Science Press*, 230 (1952).

[11] Hattersley AT, Tooke JE (1999). The fetal insulin hypothesis: An alternative explanation of the association of low bir thweight with diabetes and vascular disease. Lancet.

[12] Breschi MC, Seghieri G, Bartolomei G, Gironi A, Baldi S, Ferrannini E (1993). Relation to birthweight to maternal plasma glucose and insulin concentrations during normal pregnancy. Diabetologia.

[13] Sacks DA (2006). What proportion of birth weight is attributable to maternal glucose among infants of diabetic women?. Am. J. Obstet. Gynecol..

[14] Hughes AE (2023). Monogenic disease analysis establishes that fetal insulin accounts for half of human fetal growth. J. Clin. Invest..

[15] Hay W (2006). Fetal-placental glucose exchange and fetal glucose metabolism. Trans. Am. Clin. Climatol. Assoc..

[16] Juliusdottir T (2021). Distinction between the effects of parental and fetal genomes on fetal growth. Nat. Genet..

[17] Evans DM, Freathy RM (2021). Shedding light on the genetics of fetal growth. Nat. Genet..

[18] Beaumont RN (2018). Genome-wide association study of offspring birth weight in 86 577 women identifies five novel loci and highlights maternal genetic effects that are independent of fetal genetics. Hum. Mol. Genet..

[19] Barker D (1993). Type 2 (non-insulin-dependent) diabetes mellitus, hypertension and hyperlipidaemia (syndrome X): Relation to reduced fetal growth. Diabetologia.

[20] Hattersley A (1998). Mutations in the glucokinase gene of the fetus result in reduced birth weight. Nat. Genet..

[21] Horikoshi M (2016). Genome-wide associations for birth weight and correlations with adult disease. Nature.

[22] Tyrrell JS, Yaghootkar H, Freathy RM, Hattersley AT, Frayling TM (2013). Parental diabetes and birthweight in 236 030 individuals in the UK biobank study. Int. J. Epidemiol..

[23] Hillman S, Peebles DM, Williams DJ (2013). Paternal metabolic and cardiovascular risk factors for fetal growth restriction: a case-control study. Diabetes Care.

[24] Lindsay RS (2000). The Role of paternal inheritance in the association of low birth weight and diabetes. Diabetes.

[25] Udler MS (2018). Type 2 diabetes genetic loci informed by multi-trait associations point to disease mechanisms and subtypes: A soft clustering analysis. PLoS Med..

[26] Engelbrechtsen L (2018). Birth weight variants are associated with variable fetal intrauterine growth from 20 weeks of gestation. Sci. Rep..

[27] Scott RA (2012). Large-scale association analyses identify new loci influencing glycemic traits and provide insight into the underlying biological pathways. Nat. Genet..

[28] Dupuis J (2010). New genetic loci implicated in fasting glucose homeostasis and their impact on type 2 diabetes risk. Nat. Genet..

[29] Wood AR (2017). A Genome-wide association study of IVGTT-based measures of first-phase insulin secretion refines the underlying physiology of type 2 diabetes variants. Diabetes.

[30] Lotta LA (2017). Integrative genomic analysis implicates limited peripheral adipose storage capacity in the pathogenesis of human insulin resistance. Nat. Genet..

[31] Walford GA (2016). Genome-Wide Association Study of the modified stumvoll insulin sensitivity index identifies BCL2 and FAM19A2 as novel insulin sensitivity loci. Diabetes.

[32] Sacks DA (2004). Determinants of fetal growth. Curr. Diabetes Reports.

[33] Freathy RM (2010). Variants in ADCY5 and near CCNL1 are associated with fetal growth and birth weight. Nat. Genet..

[34] Horikoshi M (2013). New loci associated with birth weight identify genetic links between intrauterine growth and adult height and metabolism. Nat. Genet..

[35] Workalemahu T (2018). Genetic and environmental influences on fetal growth vary during sensitive periods in pregnancy. Sci. Rep..

[36] Mahajan A (2018). Fine-mapping type 2 diabetes loci to single-variant resolution using high-density imputation and islet-specific epigenome maps. Nat. Genet..

[37] Hughes AE (2018). Fetal genotype and maternal glucose have independent and additive effects on birth weight. Diabetes.

[38] Dude AM, Yee LM (2018). Identifying fetal growth disorders using ultrasonography in women with diabetes. J. Ultrasound Med..

[39] Beaumont RN (2022). Genome-wide association study of placental weight in 179,025 children and parents reveals distinct and shared genetic influences between placental and fetal growth. MedRxiv.

[40] Haeussner E, Schmitz C, von Koch F, Frank HG (2013). Birth weight correlates with size but not shape of the normal human placenta. Placenta.

[41] Burkhardt T, Schaffer L, Schneider C, Zimmermann R, Kurmanavicius J (2006). Reference values for the weight of freshly delivered term placentas and for placental weight-birth weight ratios. Eur. J. Obstet. Gynecol. Reprod. Biol..

[42] Hadlock F, Harrist R, Martinez-Poyer J (1991). In utero analysis of fetal growth—a sonographic weight standard. Radiology.

[43] Gybel-Brask D, Hogdall E, Johansen J, Christensen IJ, Skibsted L (2014). Serum YKL-40 and uterine artery Doppler—A prospective cohort study, with focus on preeclampsia and small-for-gestational-age. Acta Obstet. Gynecol. Scand..

[44] Gybel-Brask D, Johansen JS, Christiansen IJ, Skibsted L, Hogdall EV (2016). Serum YKL-40 and gestational diabetes—An observational cohort study. APMIS.

[45] Clausson B, Gardosi J, Francis A, Cnattingius S (2001). Perinatal outcome in SGA births by customised versus population-based birthweight standards. BJOG Int. J. Obstet. Gynaecol..

[46] Chang TC, Robson SC, Boys RJ, Spencer JAD (1992). prediction of the small for gestational age infant: Which ultrasonic measurement is best?. Obstet. Gynecol..

