# Supplementary material for: The influence of insulin-related genetic variants on fetal growth, fetal blood flow, and placental weight in a prospective pregnancy cohort
Source: Sci Rep. 2023 Nov 10;13:19638. doi: 10.1038/s41598-023-46910-6 (PMC10638310; doi:10.1038/s41598-023-46910-6)
Supplement: Supplementary file 1 — Supplementary Information. [file 41598_2023_46910_MOESM1_ESM.docx]

**Supplementary**

| Trait | Fasting insulin GRS (FI) | | First phase insulin release GRS (FPIR) | | Insulin resistance and dyslipidaemia GRS (IR+DLD) | | Insulin sensitivity GRS (IS) | |
| --- | --- | --- | --- | --- | --- | --- | --- | --- |
|  | β (95% CI) | P | β (95% CI) | P | β (95% CI) | P | β (95% CI) | P |
| Fetal weight (g)  (incl. birth weight) | 3.543e-05  (-5.12e-05; 1.22e-04) | 0.690 | 8.63e-05  (-1.85e-05; 1.91e-04) | **0.023** | 4.81e-06  (-4.80e-05; 5.77e-05) | 0.857 | 1.74e-04  (-9.72e-05; 4.46e-04) | 0.182 |
| Fetal weight (g)  (excl. birth weight) | 3.18e-05  (-2.39e-05; 8.74e-05) | 0.241 | 5.46e-05  (-1.30e-05; 1.22e-04) | **0.033** | 2.82e-06  (-3.10e-05; 3.66e-05) | 0.421 | 7.87e-05  (-9.62e-05; 2.54e-04) | 0.316 |
| Occipitofrontal diameter (mm) | 5.34e-07  (-1.42e-06; 2.48e-06) | 0.445 | 6.79e-07  (-1.69e-06; 3.05e-06) | 0.862 | 3.89e-07  (-8.03e-07; 1.58e-06) | 0.346 | 7.55e-06  (1.48e-06; 1.36e-05) | **0.009** |
| Biparietal diameter (mm) | 1.66e-06  (-4.70e-08; 3.37e-06) | 0.057 | 9.50e-07  (-1.13e-06; 3.03e-06) | 0.371 | 3.88e-07  (-6.60e-07; 1.44e-06) | 0.468 | 4.17e-06  (-1.17e-06; 9.52e-06) | 0.126 |
| Abdominal circumference (mm) | 2.96e-06  (-2.93e-06; 8.84e-06) | 0.324 | 6.19e-06  (-9.49e-07; 1.33e-05) | 0.089 | 8.49e-07  (-2.76e-06; 4.46e-06) | 0.645 | 8.31e-06  (-1.01e-05; 2.67e-05) | 0.377 |
| Femur length (mm) | 1.70e-07  (-8.94e-07; 1.23e-06) | 0.754 | 1.60e-06  (3.11e-07; 2.88e-06) | **0.015** | -4.01e-09  (-6.55e-07; 6.47e-07) | 0.990 | -1.17e-06  (-4.50e-06; 2.16e-06) | 0.491 |

**Table S1:** Associations between *weighted* genetic risk scores (GRS) for insulin secretion and sensitivity-related traits and fetal growth

Fetal growth was modelled using linear mixed models. Data is presented as raw parameter estimates (β), and estimates are transformed to the original scale with respective 95% confidence intervals. Beta values represent the additive per allele effect of the GRS on change in daily proportional weight gain. All traits are measured by ultrasound during pregnancy at 20, 25 and 32 weeks. Birthweight was measured at birth.

**Table S2:** Associations between *weighted* genetic risk scores (GRS) for insulin-related traits and placental weight, placental ratio, SGA/LGA, and fetal flow measures

| Trait | Fasting insulin GRS | | First phase insulin release GRS | | Insulin resistance GRS | |  |  | Insulin sensitivity GRS | |
| --- | --- | --- | --- | --- | --- | --- | --- | --- | --- | --- |
|  | β (95% CI) | P | β (SD) | P | β (SD) | P | | | β (SD) | P |
| Placental weight (g) | -2.343  (-4.546; -0.142) | **0.037** | 1.366  (-1.317; 4.050) | 0.318 | -1.442  (-3.246; 0.361) | 0.117 | | | 9.937  (3.048; 16.827) | **0.005** |
| Placental ratio  (placental weight/ birth weight) | -0.0008  (-0.0017; 0.00012) | 0.091 | -0.0005  (-0.0015; 0.0007) | 0.409 | -0.0001  (-0.0009; 0.0006) | 0.713 | | | 0.0012  (-0.0017; 0.0041) | 0.424 |
| Birth weight (g) | 7.171e-02  (-12.247; 12.390) | 0.991 | 14.863 (3.710e-02; 29.688) | **0.049** | 0.765  (-1.901; 3.431) | 0.575 | | | 22.074  (-16.370; 60.518) | 0.206 |
| Small for gestational age fetus | -0.0033  (-0.1052; 0.0986) | 0.950 | -0.0063  (-0.1301; 0.1174) | 0.920 | 0.0376  (-0.0464; 0.1216) | 0.381 | | | 0.0112  (-0.3094; 0.3317) | 0.946 |
| Large for gestational age fetus | -0.0469  (-0.2049; 0.1110) | 0.560 | -0.1025  (-0.2897; 0.0846) | 0.283 | -0.0612  (-0.1823; 0.0598) | 0.321 | | | 0.1346  (-0.3151; 0.5842) | 0.558 |
| Umbilical flow (PI) | 0.0015  (-0.0020; 0.0050) | 0.407 | -0.0001  (-0.0043; 0.0041) | 0.960 | 0.0021  (-0.0007; 0.0050) | 0.138 | | | -0.0030  (-0.0138; 0.0077) | 0.580 |
| Middle cerebral artery flow (PI) | 0.0010  (-0.0118; 0.0139) | 0.876 | -6.23e-03  (-0.0215; 0.0091) | 0.424 | -6.26e-03  (-0.0171; 0.0046) | 0.259 | | | 3.19e-03  (-0.0377; 0.0441) | 0.878 |

Placental weight, placental ratio, and birthweight were modelled using linear regression. SGA and SGA were modelled using logistic regression. Flow parameters were modelled using generalized linear regression. Data are presented as raw parameter estimates (β), and estimates are transformed to the original scale with respective 95% confidence intervals. SGA indicates a birthweight less than 10^th^ percentile for the gestational age. LGA indicates a birthweight above the 90^th^ percentile for the gestational age.

**Figure S1:** Flowchart of women included in the pregnancy cohort


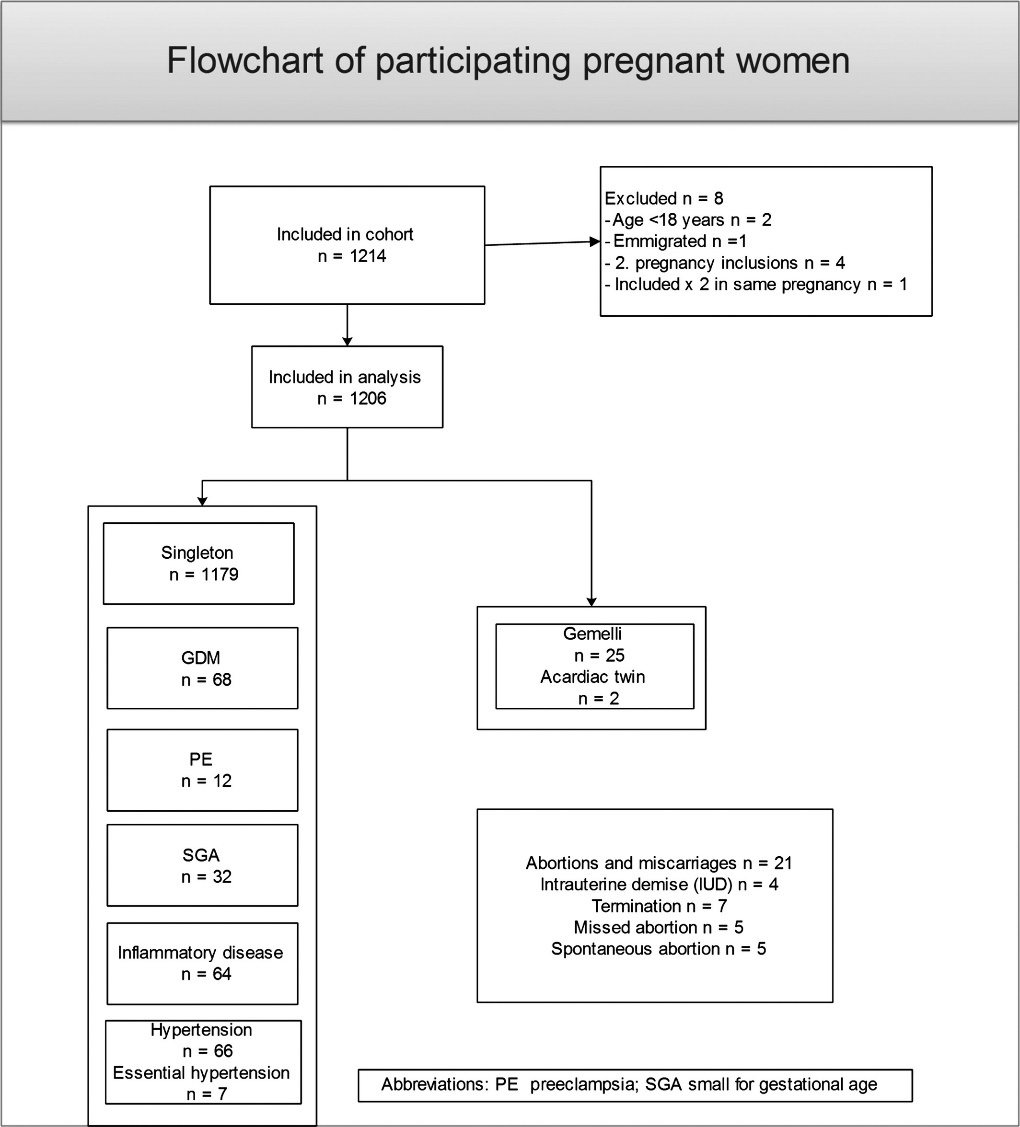


A total of 1179 singleton pregnant women were included in the final cohort. The above figure provides an overview of the exclusion of study participants as well as pregnancy complications within the final cohort. Figure adapted from Gybel-Brask, D., Hogdall, E., Johansen, J., Christensen, I. J. & Skibsted, L. Serum YKL-40 and uterine artery Doppler -- a prospective cohort study, with focus on preeclampsia and small-for-gestational-age. *Acta Obstet Gynecol Scand* **93**, 817-824 (2014).
